# Retraction Note: Enhanced Emission of Quantum System in Si-Ge Nanolayer Structure

**DOI:** 10.1186/s11671-018-2454-0

**Published:** 2018-02-07

**Authors:** Zhong-Mei Huang, Wei-Qi Huang, Tai-Ge Dong, Gang Wang, Xue-Ke Wu

**Affiliations:** 1 State key laboratory of Surface Physics, Key Laboratory of Micro and Nano Photonic Structures (Ministry of Education) and Department of Physics, Fudan University, Shanghai, 200433 China; 2 Institute of Nanophotonic Physics, Guizhou University, Guiyang, 550025 China

## Retraction Note

The Editor has retracted this article [1] due to significant overlap in text and figures with a previous article published in another journal [2]. The authors do not agree with the retraction.

[1] Huang Z-M et al. (2016) Enhanced Emission of Quantum System in Si-Ge Nanolayer Structure. *Nanoscale Res. Lett.* 11:462; first published 18 October 2016.

[2] Huang Z-M et al. (2017) Enhanced emission in the three-level system of Si and Ge nanostructures. *Optics Commun.* 383:1-5; first published 30 August 2016.
